# Mechanism of Selective VEGF-A Binding by Neuropilin-1 Reveals a Basis for Specific Ligand Inhibition

**DOI:** 10.1371/journal.pone.0049177

**Published:** 2012-11-08

**Authors:** Matthew W. Parker, Ping Xu, Hou-Fu Guo, Craig W. Vander Kooi

**Affiliations:** Department of Molecular and Cellular Biochemistry and Center for Structural Biology, University of Kentucky, Lexington, Kentucky, United States of America; Imperial College London, United Kingdom

## Abstract

Neuropilin (Nrp) receptors function as essential cell surface receptors for the Vascular Endothelial Growth Factor (VEGF) family of proangiogenic cytokines and the semaphorin 3 (Sema3) family of axon guidance molecules. There are two Nrp homologues, Nrp1 and Nrp2, which bind to both overlapping and distinct members of the VEGF and Sema3 family of molecules. Nrp1 specifically binds the VEGF-A_164/5_ isoform, which is essential for developmental angiogenesis. We demonstrate that VEGF-A specific binding is governed by Nrp1 residues in the b1 coagulation factor domain surrounding the invariant Nrp C-terminal arginine binding pocket. Further, we show that Sema3F does not display the Nrp-specific binding to the b1 domain seen with VEGF-A. Engineered soluble Nrp receptor fragments that selectively sequester ligands from the active signaling complex are an attractive modality for selectively blocking the angiogenic and chemorepulsive functions of Nrp ligands. Utilizing the information on Nrp ligand binding specificity, we demonstrate Nrp constructs that specifically sequester Sema3 in the presence of VEGF-A. This establishes that unique mechanisms are used by Nrp receptors to mediate specific ligand binding and that these differences can be exploited to engineer soluble Nrp receptors with specificity for Sema3.

## Introduction

The Nrp family of receptors coordinate ligand-binding events that mediate endothelial cell migration and proliferation and neuronal chemorepulsion (reviewed in [1]). There are two Nrp homologues, Nrp1 and Nrp2, which share the same overall domain architecture with 44% identity in their primary sequence. Nrp ligands include the VEGF family of pro-angiogenic cytokines [2] and the Sema3 family [3], [4] of axon guidance molecules [5]. Both VEGF and Sema3 family of ligands are composed of multiple genes, splice forms, and proteolytic products with different receptor binding specificity and physiological function.

Soluble receptors capable of sequestering specific ligands are an attractive modality for blocking ligand-dependent signaling pathways. VEGF-Trap, a soluble chimera of VEGF receptor (VEGFR), containing the domains necessary for ligand binding [6], has been approved for use as a clinical agent blocking VEGF-A dependent angiogenesis [7]. The identification of endogenously expressed soluble Nrp receptors (sNrp) with anti-tumor activity [8] has prompted interest in the use of engineered Nrp molecules as specific pathway inhibitors. It was recently reported that mutation to the Nrp2-b1 domain enhances its ability to bind VEGF-A [9] and that administration of this truncated receptor effectively antagonizes VEGF-A dependent angiogenic signaling [10]. A Sema3 specific inhibitor would be of significant utility. While Sema3 mediated repulsive cues are essential during development, they pose a significant barrier to axonal regrowth following injury [11]. This is particularly the case in repair following spinal cord injury. In response to spinal cord injury a glial scar forms that serves as a barrier to regenerating axons. Sema3 family members are produced by meningeal cells located in the glial scar and are a major component of the repulsive cues that prevent axonal regeneration [12], [13]. Blocking inhibitory cues represents one fundamental mode of regenerative therapy for partial cord injuries [14]. However, due to limited understanding of the determinants of Nrp binding specificity, no soluble Nrp-trap exists that is specific for Sema3.

The conserved Nrp architecture provides Nrp homologues with the ability to bind ligands using a common binding mode. The b1 coagulation factor domain of Nrp1 and Nrp2 contains a conserved cleft optimally suited for binding a C-terminal arginine that is necessary for ligand binding [15]–[17]. All VEGF family members contain a C-terminal arginine and all Sema3 family members contain at least one conserved furin recognition sequence that is endogenously cleaved to liberate a C-terminal arginine [18], [19]. Indeed, binding to this shared site underlies the observed competition between VEGF and Sema3 [9], [19].

In physiological context, physical and functional specificity is observed between receptor-ligand pairs. Nrp1 acts as the functional receptor for VEGF-A [2] and Sema3A [3], [4] and Nrp2 acts as the functional receptor for VEGF-C [20] and Sema3F [21]. The mechanism underlying specific Sema3 family member binding by Nrp1 and Nrp2 has been shown to involve dual-site binding. The Nrp b1 domain binding to the Sema3 C-terminal domain is necessary for high-affinity binding but does not display specificity for Nrp1 or Nrp2 [19], [21]. Secondarily, the N-terminal a1 domain of Nrp1 and Nrp2 selectively binds the sema domain of different Sema3 family members [22]–[24]. Indeed, a Sema3 binding-deficient Nrp has been reported which disrupts the unique a1/sema interaction [25]. However, since there is no known secondary binding site, the basis for specificity in VEGF binding remains unclear.

It was recently demonstrated that the essential VEGF-A_164/165_ (VEGF-A) isoform binds preferentially to Nrp1 [16]. The C-terminal arginine binding cleft of the Nrp1-b1 domain is formed by three loops which are a common feature among coagulation factor domains. While the cleft is conserved, a significant number of residues surrounding this binding pocket differ between Nrp1 and Nrp2 and may contribute to the observed ligand binding specificity. Indeed, the L1 loop of the Nrp b1 domain has been shown to contribute to the observed preferential binding of VEGF-A to Nrp1 [16]. However, this interaction alone is insufficient to explain the marked difference in potency of VEGF-A binding to Nrp1 and Nrp2, therefore other molecular determinants of this preferential binding must exist. The recently reported Nrp2 mutation, when Nrp2 R287 is replaced with the corresponding Nrp1 E285, shows enhanced VEGF-A binding and, importantly, unchanged binding to Sema3F [9]. Together, these data suggest that the Nrp b1 domain contains distinct features that govern specific ligand binding and could be exploited to produce specific inhibitors of Nrp ligands. By understanding how selective VEGF binding is achieved, and whether these regions also affect Sema3 binding, a Nrp molecule that specifically bound Sema3 and blocked Sema3 repulsive cues could be designed as a potentially important therapeutic tool.

In the present study we determined the basis for preferential Nrp1 binding to VEGF-A. Mutagenesis of residues surrounding the shared C-terminal arginine binding pocket identifies residues that differ between Nrp1 and Nrp2 and underlie the observed VEGF-A specificity. A chimeric Nrp2, which combines the identified mutations, is capable of binding VEGF-A similarly to Nrp1 whereas a chimeric Nrp1 shows significant loss of VEGF-A binding. We further show that Nrp1 and Nrp2 both bind Sema3F with similar affinity and that both Nrp2 and the chimeric Nrp1 can selectively sequester Sema3. These data establish that unique mechanisms are used by Sema3 and VEGF-A to mediate specific Nrp binding, revealing a basis for the use of engineered Nrp molecules as selective inhibitors of Sema3.

## Materials and Methods

### Protein Expression and Purification

The high affinity ligand binding b1b2 domains of human Nrp1 and Nrp2, and VEGF-A_165_ were expressed in *Escherichia coli,* as previously reported, and purified according to established procedures [15], [19], [26]. Chimeric Nrp1 and Nrp2 constructs were generated using the megaprimer method, expressed as a 6XHis-tag fusion from pET28, and purified using immobilized metal affinity chromatography (IMAC) followed by heparin agarose affinity, as with wild-type proteins. Proteins were buffer exchanged into binding buffer (20 mM Tris pH 7.5, 50 mM NaCl).

Large-scale transient transfection of Chinese Hamster Ovary (CHO) cells was used to express alkaline phosphatase (AP) fusion proteins from pAPtag-5 (GenHunter, Nashville, TN) [19], [27]. AP-Nrp1 and AP-Nrp2 b1b2 mutants were generated using the megaprimer method. All AP-Nrp wild-type and mutant proteins were produced at similar levels. AP-Nrp conditioned media was concentrated and buffer exchanged into binding buffer. All expression plasmids and mutants were verified by DNA sequencing.

### AP-VEGF-A Inhibition Assay

Nrp affinity plates were prepared by adding 100µL of Nrp1 diluted 1/10 in 50 mM Na_2_CO_3_ pH 10.4 to a final concentration of 2.5 ng/µl in 96 well high-binding plates (Costar, 9018), incubated for 1 hr at 37°C, washed with PBS-T (PBS, 0.1% Tween20), and stored at 4°C. An AP-fusion of VEGF-A_164_ (AP-VEGF-A) was utilized for VEGF inhibition experiments [19]. Nrp1 and Nrp2 were combined with AP-VEGF-A (155 µmole p-NPP hydrolyzed/min/L) in incubation buffer (20 mM Tris pH 7.5, 300 mM NaCl) to a final volume of 100µL and incubated with the Nrp1 affinity plates for 1 hr at 25°C. Wells were washed three times with PBS-T using an EL404 plate washer (BioTek, Winooski, VT), and incubated with PBS-T for an additional 5 min. Wash solution was removed, and 100µL of 1× AP Assay Reagent [28] was added. The reaction was quenched by addition of 100µL of 0.5 N NaOH. Evolved p-nitrophenol (p-NP) was quantitated at 405 nm using a SpectraMax M5 plate reader (Molecular Devices, Sunnyvale, CA). The IC_50_ was fit with a nonlinear dose-response inhibition curve using Prism (Graphpad Software, La Jolla, CA). The inhibitory potency of Nrp1^Chimera^ and Nrp2^Chimera^ was determined using the same method.

### AP-Nrp Binding Assay

To assess the role of the Nrp coagulation factor loops in VEGF-A binding, we measured AP-Nrp binding to VEGF-A affinity plates. Affinity plates were prepared by adding 100µL of VEGF-A_165_ diluted 1/10 in 50 mM Na_2_CO_3_ pH 10.4 to a final concentration of 50 ng/µl in 96 well plates. AP-Nrp constructs (724 µmole p-NPP hydrolyzed/min/L) were incubated with VEGF-A affinity plates for 1 hr at 25°C. Plates were washed and retained AP activity quantitated as described above.

### AP-Sema3F and AP-Sema3F-Ig-basic Inhibition Assay

Two AP-fusions of Sema3F were prepared, one corresponding to full length Sema3F (AP-Sema3F; residues 20–779) and the other an AP-fusion of the Sema3F Ig and basic domains (AP-Sema3F-Ig-basic; residues 605–779), the domains necessary and sufficient for binding the b1 coagulation factor domain of Nrp [22]. These were used for semaphorin inhibition experiments by Nrp. Nrp1 and Nrp2 were combined with AP-Sema3F (648 µmole p-NPP hydrolyzed/min/L) in incubation buffer and incubated in low density Nrp1 affinity plates for 1 h at 25°C. Similarly, Nrp1 and Nrp2 were combined with AP-Sema3F-Ig-basic (181 µmole p-NPP hydrolyzed/min/L) in binding buffer and incubated in high density Nrp1 affinity plates for 1 hr at 25°C. High density Nrp1 affinity plates were prepared by adding 100µL of Nrp1 diluted 1/10 in 50 mM Na_2_CO_3_ pH 10.4 to a final concentration of 50 ng/µl in 96 well plates. Plates were washed and retained AP activity quantitated as described above.

### C-furSema Inhibition Assay

Two peptides corresponding to the C-terminal basic domains of furin processed Sema3A (C-furSema3A; residues 718–769) (Neo-Peptide, Cambridge, MA) and Sema3F (C-furSema3F; residues 740–779 [19]) (LifeTein, South Plainfield, NJ) were synthesized, oxidized to form the natural inter-molecular disulfide, and purified. Peptides were resuspended and titrated with AP-VEGF-A (100 µmole p-NPP hydrolyzed/min/L) in incubation buffer and added to low density Nrp1 affinity plates for 1 h at 25°C. Plates were washed and retained AP activity quantitated as described above.

### AP-VEGF-A Recovery Assay

To measure the preferential binding of Nrp1 and Nrp2 to Sema3F or VEGF-A the potential of Nrp to promote the recovery of C-furSema [19] mediated inhibition of AP-VEGF-A binding to Nrp1 was assessed. Nrp1 and Nrp2 were combined with both AP-VEGF-A (155 µmole p-NPP hydrolyzed/min/L) and C-furSema3F (45 nM, the concentration at which ≈90% inhibition is achieved) in incubation buffer and added to low density Nrp1 affinity plates for 1 hr at 25°C. Plates were washed and retained AP activity quantitated as described above. Retained AP-VEGF-A was reported versus Nrp concentration as the percent of binding observed relative to uninhibited AP-VEGF-A (no C-furSema).

### Circular Dichroism

Circular dichroism (CD) spectra were collected using a Jasco J-810 Spectropolarimeter. Wild-type and chimeric proteins were added to a 0.1-cm-pathlength cuvette at 10µM in PBS pH 7.4. Spectra were recorded at a speed of 20 nm/min using the average of three accumulations over a range of 205–245 nm. Data are reported as per residue molar ellipticity.

## Results

### Soluble Nrp1 Selectively Inhibits VEGF-A

**Figure 1:**
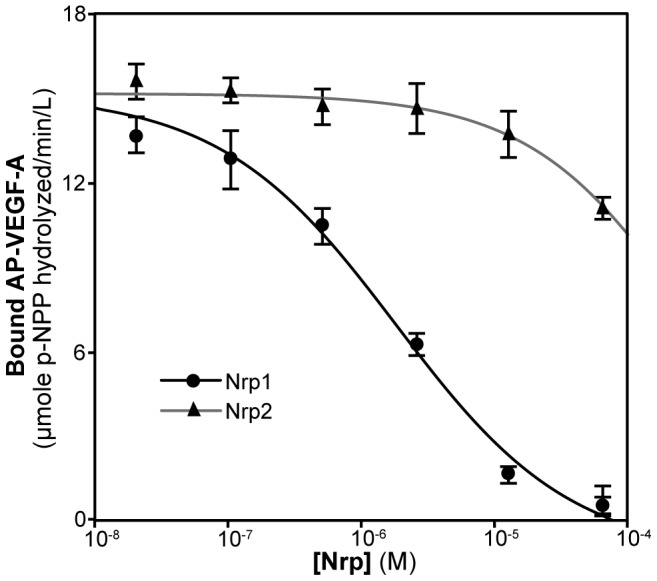
Nrp1 selectively inhibits VEGF-A binding. The ability of Nrp1 and Nrp2 to selectively sequester AP-VEGF-A from Nrp1 adsorbed on affinity plates was assessed. The amount of retained AP-VEGF-A was measured and the Nrp concentration recorded where half-inhibition was achieved. Nrp1 inhibited the binding of VEGF-A with an IC_50_ = 1.8 µM (black line). Inhibition by Nrp2 was seen only at the highest concentration of protein attainable with an estimated IC_50_≈310 µM (grey line). Experiments were performed in triplicate and reported as the mean ±1 S.D.

To assay the inhibitory potency of Nrp1 and Nrp2, we tested the ability of Nrp1 and Nrp2 to inhibit VEGF-A binding to Nrp1 affinity plates in a dose dependent manner (Figure 1). Nrp1 was able to potently inhibit the binding of VEGF-A to Nrp1 affinity plates with an IC_50_ = 1.8 µM (log IC_50_ = −5.7±0.2) (black line, Figure 1). In contrast, Nrp2 was able to inhibit binding only at the highest concentrations with an IC_50_≈310 µM (log IC_50_ = −3.5±0.4) (grey line, Figure 1). Each experiment was performed in triplicate with unique protein preparations to provide a direct measurement of inter-assay variability, with 11% inter-assay variability observed for Nrp1 compared to 3.5% intra-assay variability (Figure S1). These data demonstrate, both qualitatively and quantitatively, the robust quality and reproducibility of the reported data. The greater than 100-fold difference in IC_50_ between Nrp1 and Nrp2 is consistent with the previously reported VEGF binding selectivity [16].

### Identification of the Regions of Nrp1 that Confer Selective VEGF-A Binding

**Figure 2:**
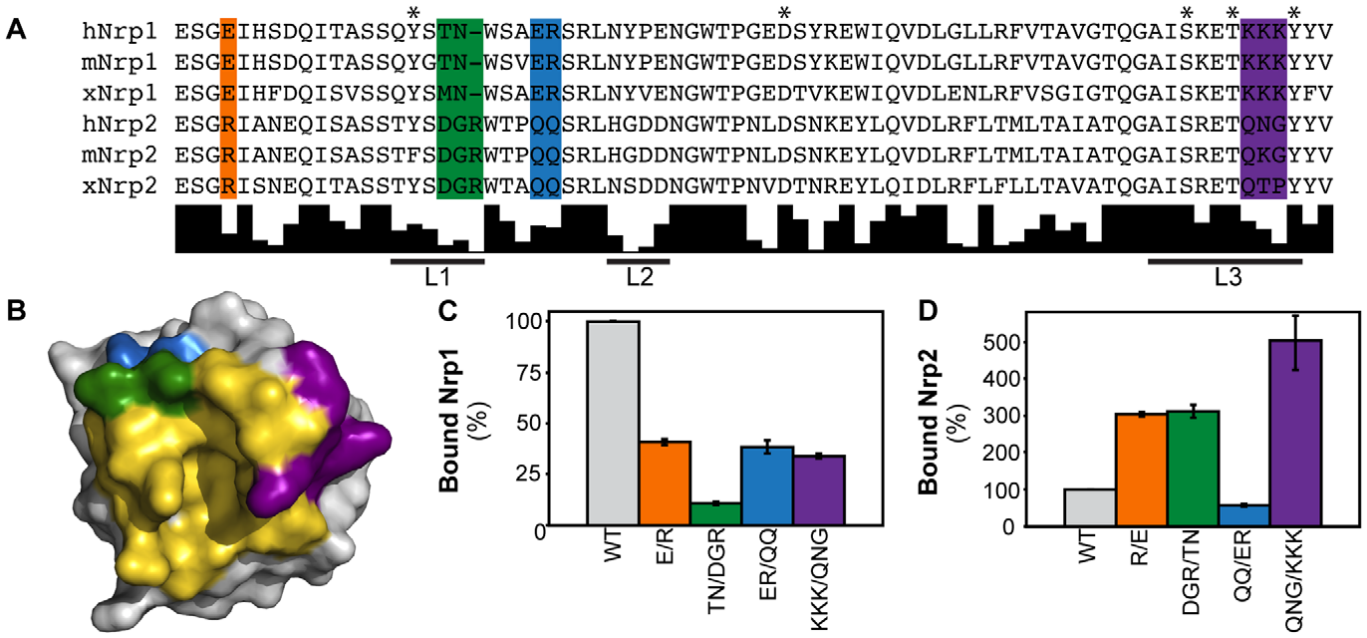
Nrp1 residues mediate specific VEGF-A binding. (A) Alignment of orthologous Nrp1 and Nrp2 b1 domains shows conservation of residues critical for C-terminal arginine binding (marked with a *) but variability within regions surrounding the interloop cleft (orange: Nrp1:E285/Nrp2:R287 [9]; green: Nrp1∶299-TN-300/Nrp2∶301-DGR-303; blue: Nrp1∶304-ER-305/Nrp2∶307-QQ-308; purple: Nrp1∶350-KKK-352/Nrp2∶353-QNG-355). Below the alignment is a conservation histogram illustrating identity across the displayed sequences. (B) Surface representation of the Nrp b1 domain reveals that the direct VEGF-A binding region (gold) [16] is closely associated with the selected regions (colored according to 2A) in three-dimensional space. (C) VEGF-A binding of Nrp1 mutants reveals loss of binding for each mutant protein compared to wild-type. Retained AP-Nrp1 binding is reported as the percent of retained wild-type AP-Nrp1. (D) Determination of the VEGF-A binding capacity of Nrp2 mutants reveals that three of the four Nrp2 chimeras show enhanced VEGF-A binding compared to wild-type. Retained AP-Nrp2 binding is reported as the percent of retained wild-type AP-Nrp2. Experiments were performed in triplicate and reported as the mean ±1 S.D.

The selective ability of Nrp1 to inhibit VEGF-A binding led us to consider the mechanism of this specificity. The predominant Nrp structural determinants mediating VEGF-A binding have been localized to domain b1 [9], [16], [25]. The b1 domains of Nrp1 and Nrp2 are 52% identical. Conserved between both are the residues which form the C-terminal arginine binding pocket demonstrated to be essential for VEGF-A/Nrp binding [15] (Figure 2A, asterisk). Alignment of Nrp1 and Nrp2 b1 domains reveals diversity in the primary sequence of residues surrounding the binding pocket. There are three different categories of residues: those that are well conserved in both Nrp1 and Nrp2, those that are not well conserved, and those which are distinct between Nrp1 and Nrp2 but well conserved within each ortholog. We hypothesized that residues in this last category likely underlie the observed specific Nrp1/VEGF-A binding. Further, residues nearby the binding pocket, especially those in the coagulation-factor loops (L1–L3) often utilized in ligand binding in coagulation-factor domain proteins [29], most likely underlie specific binding.

Based on these two criteria, i.e., that residues are separately conserved within Nrp1 and Nrp2 and that they are nearby the binding site, four regions were selected: Nrp1:E285/Nrp2:R287, Nrp1∶299-TN-300/Nrp2∶301-DGR-303, Nrp1∶304-ER-305/Nrp2∶307-QQ-308, and Nrp1∶350-KKK-352/Nrp2∶353-QNG-355 (Figure 2B). Mutant proteins were produced by swapping the selected sequences between the two Nrp homologues. Nrp residues that contribute to the observed specificity would be expected to reduce Nrp1 binding and enhance Nrp2 binding when reversed. These chimeric Nrp molecules were expressed with an N-terminal AP tag. The binding of AP-Nrp constructs to VEGF-A affinity plates was measured and normalized relative to wild-type Nrp1 or Nrp2 binding. Reduced binding to VEGF-A was observed for all Nrp1 chimeras relative to wild-type Nrp1 (Figure 2C), suggesting a role of these sequences in VEGF-A binding. Three of the four Nrp2 chimeras showed enhanced affinity for VEGF-A relative to wild-type Nrp2 (Figure 2D). The reduction in VEGF-A binding for both Nrp1 ER/QQ and Nrp2 QQ/ER suggests that these mutations may destabilize the protein and this mutation was therefore excluded from further study. These results suggest that E285, 299-TN-300, and 350-KKK-352 of the Nrp1-b1 domain contribute to selective VEGF-A binding.

### Nrp2^Chimera^ Inhibits VEGF-A Binding

**Figure 3:**
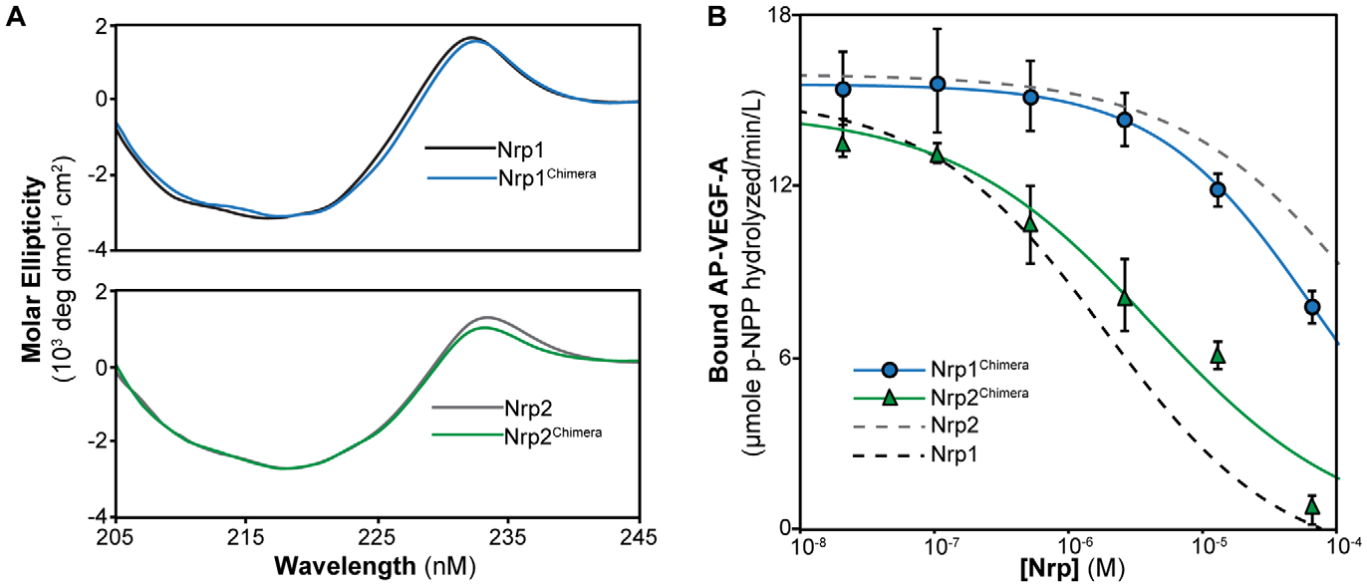
Nrp^Chimera^ molecules exhibit reversed VEGF-A specificity. (A) The secondary structure of WT Nrp and Nrp^Chimera^ was assessed by CD. The overlapping spectra of Nrp^Chimera^ with wild-type Nrp demonstrate that the incorporated mutations are not structurally deleterious. (B) Nrp1^Chimera^ (blue line) and Nrp2^Chimera^ (green line) were tested for their ability to selectively sequester AP-VEGF-A from Nrp1 adsorbed on affinity plates. The Nrp^Chimera^ molecules show reversed VEGF-A specificity with Nrp1^Chimera^ having a marked reduction in inhibitory potency (IC_50_ = 62 µM) and Nrp2^Chimera^ exhibiting a significant gain in potency (IC_50_ = 3.9 µM). Wild-type Nrp1 (black dotted line, IC_50_ = 1.8 µM) and Nrp2 (grey dotted line, IC_50_≈310 µM) are shown for comparison (data from Figure 1). Experiments were performed in triplicate and reported as the mean ±1 S.D.

To assess whether the identified loops compose the predominant features of the Nrp1-b1 domain that confer specific VEGF-A binding, we generated a Nrp1 and Nrp2 molecule which incorporated all three different mutations in a single construct, termed Nrp1^Chimera^ and Nrp2^Chimera^. To ensure that the mutant proteins were well folded, CD was utilized to assess wild-type and chimeric constructs (Figure 3A). All proteins produce spectra consistent with the expected β-sandwich architecture of the protein. Further, wild-type and mutant proteins show superimposable spectra demonstrating that the mutations are not structurally deleterious. As a quantitative measure of the selectivity of Nrp^Chimera^ proteins for VEGF-A, we assayed the potency of Nrp1^Chimera^ and Nrp2^Chimera^ in inhibiting AP-VEGF-A binding to Nrp1 affinity plates (Figure 3B). The dose-dependent ability of these constructs to inhibit VEGF-A binding was compared to wild-type Nrp1 and Nrp2 (Figure 1). The Nrp1^Chimera^ showed a significant loss in potency, with an IC_50_ = 62 µM (log IC_50_ = −4.2±0.1) (blue line, Figure 3B), an over 30-fold loss in potency compared to Nrp1 (black line, Figure 1). Strikingly, Nrp2^Chimera^ gained over 70-fold potency relative to Nrp2, with an IC_50_ = 3.9 µM (log IC_50_ = −5.4±0.2) (green line, Figure 3B), nearly to the level observed for wild-type Nrp1.

### Nrp1 and Nrp2 Equivalently Inhibit Sema3F Binding

**Figure 4:**
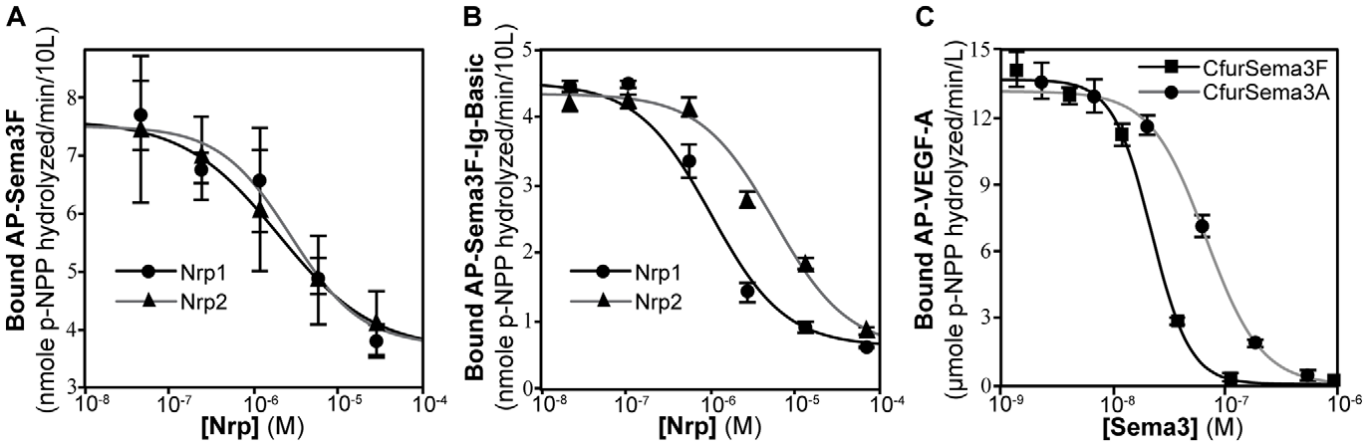
Nrp inhibits Sema3F binding through interaction with the C-terminal basic domain. (A) Nrp1 and Nrp2 dependent inhibition of AP-Sema3F binding to Nrp1 affinity plates was measured. Both Nrp homologues showed similar ability to compete for AP-Sema3F, with Nrp1 inhibiting with an IC_50_ = 2.0 µM and Nrp2 with an IC_50_ = 2.7 µM. (B) The ability of Nrp1 and Nrp2 to selectively sequester AP-Sema3F-Ig-basic from Nrp1 adsorbed on affinity plates was assessed. The amount of retained AP-Sema3F-Ig-basic was measured and the Nrp concentration recorded where half-inhibition was achieved. Nrp1 (black line) and Nrp2 (grey line) had similar ability for inhibiting Sema3F binding with IC_50_ = 1.2 µM and IC_50_ = 6.2 µM, respectively. (C) Two peptides corresponding to the C-terminal basic domain of Sema3A (C-furSema3A) and Sema3F (C-furSema3F) were analyzed for their ability to inhibit AP-VEGF-A binding to Nrp1 affinity plates. Both peptides potently inhibited binding with an IC_50_ = 22 nM and 67 nM for C-furSema3F and C-furSema3A, respectively. Experiments were performed in triplicate and reported as the mean ±1 S.D.

VEGF and semaphorin are the two major ligand families of Nrp receptors. The Sema3 family of ligands has been demonstrated to bind Nrp receptors via a dual-site binding mechanism. The Sema3 semaphorin domain mediates specific ligand binding via the Nrp a1 domain [23] and the Sema3 basic C-terminus mediates common high-affinity binding to the Nrp b1b2 domains [25]. To confirm that the Nrp1 and Nrp2 b1b2 domains bind to Sema3, and that they do not display specificity, we measured their ability to inhibit the binding of AP-Sema3F to Nrp1. Both Nrp1 and Nrp2 showed a dose-dependent inhibition of AP-Sema3F binding with equivalent observed potency for Nrp1, IC_50_ = 2.0 µM (log IC_50_ = −5.7±0.1), and Nrp2, IC_50_ = 2.7 µM (log IC_50_ = −5.6±0.3) (Figure 4A). To confirm that this interaction is mediated by the C-terminal domains of Sema3, we assayed the ability of Nrp1 and Nrp2 b1b2 domains to inhibit the binding of AP-Sema3F-Ig-basic to Nrp1. Consistent with the potency against full-length Sema3F, Nrp1 inhibited AP-Sema3F-Ig-basic binding with an IC_50_ = 1.2 µM (log IC_50_ = −5.9±0.1) and Nrp2 inhibited AP-Sema3F-Ig-basic binding with an IC_50_ = 6.2 µM (log IC_50_ = −5.2±0.2) (Figure 4B).

These data demonstrate that the b1b2 domain of Nrp1 and Nrp2 contain structural determinants capable of C-terminal Sema3F binding and that this binding does not show specific binding to the two Nrp homologues. To confirm that the interaction between the basic domain of Sema3F and Nrp1-b1b2 is conserved across the Sema3 family, and that this interaction site overlaps with that for VEGF-A, we measured the ability of a peptide corresponding to the C-terminus of both Sema3F and Sema3A to inhibit VEGF-A binding. C-furSema3F and C-furSema3A, peptides corresponding to the furin-activated forms of Sema3F and Sema3A, respectively, were assayed for their ability to competitively inhibit the binding of VEGF-A. Both peptides showed potent, dose-dependent inhibition of AP-VEGF-A binding with IC_50_ = 22 nM (log IC_50_ = −7.7±0.04) and 67 nM (log IC_50_ = −7.2±0.04) for C-furSema3F and C-furSema3A, respectively. These data confirm that the Sema3 family of ligands utilize their basic C-terminus for equivalent high-affinity binding to the Nrp b1b2 domains, and that this binding is competitive with that of VEGF-A.

### Nrp2 and Nrp1^Chimera^ Relieve C-furSema Mediated Inhibition of AP-VEGF-A Binding to Nrp1

**Figure 5:**
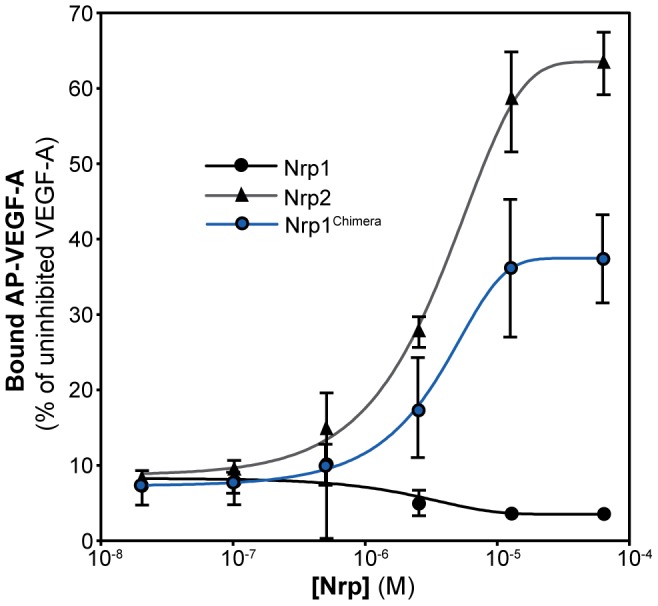
Nrp2 and Nrp1^Chimera^ preferentially sequester Sema3F. The ability of Nrp1, Nrp2, and Nrp1^Chimera^ to selectively sequester C-furSema was determined through combined incubation of Nrp, C-furSema3F, and AP-VEGF-A in Nrp1 adsorbed affinity plates. Nrp1 was unable to relieve C-furSema-dependent inhibition and completely abolished VEGF-A binding (black line). Conversely, titration with Nrp2 promoted 63% recovery of VEGF-A binding demonstrating selective sequestration of Sema3F (grey line). Nrp1^Chimera^ also relieved C-furSema-dependent inhibition, promoting recovery of VEGF-A binding to 37% the level of uninhibited VEGF-A (blue line). Experiments were performed in triplicate and reported as the mean ±1 S.D.

Nrp1 and Nrp2 display similar affinity for Sema3F (Figure 4A and 4B) but Nrp2 has markedly reduced binding to VEGF-A relative to Nrp1 (Figure 1). This data suggests that Nrp2 may be a potent and specific inhibitor of Sema3 binding to Nrp receptors. To demonstrate the use of Nrp2 as a selective semaphorin inhibitor, we assayed the ability of Nrp2 to selectively relieve Sema3-dependent inhibition of VEGF-A binding. Selectivity would be demonstrated by a reduction of C-furSema mediated-inhibition and resultant gain in AP-VEGF-A binding to Nrp1 affinity plates. Nrp1 showed no ability to relieve the inhibition of Sema3F-mediated inhibition. In fact, Nrp1 directly sequestered VEGF-A at high Nrp concentrations resulting in complete loss of binding as expected (black line, Figure 5). Remarkably, Nrp2 significantly enhanced the amount of VEGF-A retained on Nrp1 plates to 63% the level of retention seen in the absence of inhibition (grey line, Figure 5). This provides direct evidence that Nrp2 is able to directly and specifically sequester Sema3. Similarly, 37% recovery was seen with Nrp1^Chimera^ (blue line, Figure 5), consistent with the reversal of specificity seen for VEGF-A inhibition (Figure 3B). These data demonstrate that the unique mechanisms utilized by Nrp to preferentially bind different members of the Sema3 and VEGF family ligands can be exploited to create Nrp inhibitors specific for different Nrp ligand families.

## Discussion

These data establish the basis for selective Nrp ligand binding to the b1 coagulation factor domain of Nrp. While both VEGF and Sema3 ligand families share a partially overlapping ligand binding site in the Nrp b1 domain [19], we demonstrate a series of residues that differ between Nrp1 and Nrp2 and contribute to the observed specific VEGF-A binding.

Nrp2^Chimera^ possesses nearly full reversal of selectivity to the level of Nrp1, indicating that the three altered regions represent the predominant regions mediating specific VEGF-A binding. The TN/DGR L1-loop is highly divergent between Nrp1 and Nrp2 and possesses the only insertion/deletion in the b1 domain. Mutation of this region results in the largest net loss of VEGF-A binding to Nrp1, consistent with a direct interaction involving a hydrogen bond between Nrp1-T299 and VEGF-A-E154 [16]. The KKK/QNG L3-loop produces the largest net gain in Nrp2 binding to VEGF-A. Conserved residues in the L3 loop form one wall of the C-terminal arginine binding pocket. The non-conserved KKK/QNG residues do not appear to directly engage VEGF-A in the bound form. The residues are, however, very distinct in physical properties with the Nrp1 loop being significantly more electropositive. VEGF-A contains a series of conserved acidic residues in the C-terminus of its heparin-binding domain, including E154 discussed above, which contribute to Nrp binding. This suggests that the L3-loop may function by electrostatic steering of VEGF-A. This may represent a general mechanism allowing specific binding of other Nrp1 specific ligands such as VEGF-B and placental growth factor (PlGF) that also possess acidic residues in their heparin-binding domains. In contrast, Sema3 family members and Nrp2-specific VEGF-C and VEGF-D do not possess these acidic residues. A mutant Nrp2 containing R287E has previously been shown to possess enhanced binding to VEGF-A [9]. We show that charge reversal produces a decrease in Nrp1 binding to VEGF-A indicating a direct contribution to ligand binding selectivity. Geretti and colleagues proposed that this may be due to enhancing the electronegative potential of the b1 domain of Nrp2 favoring binding to the electropositive VEGF-A. R287 is located in a helical region directly adjacent to the L1 loop, suggesting that these regions interact to correctly position these two structural elements, and thus the critical ligand binding coagulation factor loops. While the Nrp^Chimera^ molecules substantially reverse the observed specificity for VEGF-A, the reversal does not quite reach the corresponding wild-type level and one or more additional regions may also contribute to selective binding.

Through understanding the molecular basis for Nrp’s specific binding of its ligands, engineered soluble Nrp receptors can be designed with specificity for a particular ligand. The binding of Sema3F shows little selectivity between Nrp1 and Nrp2. Further, the observed inhibitory potency of Nrp1 for VEGF-A and Sema3F is virtually identical in absolute terms. Thus, Nrp1 was found to be equally capable of sequestering both VEGF-A and Sema3F and suggests that Nrp1 has utility as a broad-spectrum Nrp ligand inhibitor. In contrast, Nrp2 is found to selectively sequester Sema3F. The reported chimeric Nrps have utility for discriminating between the contribution of VEGF-A and Sema3F function in a particular system. Previous work has reported Nrp1 mutations in the a1 domain that allow production of a VEGF-A selective Nrp1 [25]. The Nrp1^Chimera^ reported here represents a complimentary molecule that is selective for Sema3F, with utility in differentiating between specific effects mediated by the different Nrp1 ligands.

The use of soluble Nrp as a modulator of VEGF-mediated angiogenesis has been the target of numerous studies. Soluble Nrp as a modality for blocking the chemorepulsive function of Sema3 is less established even though clear clinical applications exist for neutralizing molecules in spinal cord injury [30]. The efficacy of targeting this signaling axis is demonstrated by a small molecule inhibitor of the Sema3A/Nrp1 interaction [31] that, when given to rats following spinal cord transection, shows enhanced regeneration of axons across the glial scar [32]. Two outstanding issues remain to be solved. First, many members of the Sema3 family can mediate this deleterious axonal repulsion, so a potent pan-Sema3 inhibitory modality is desired. Second, selective inhibition of Sema3 signaling without effecting VEGF-A signaling is desired to maximally promote recovery following injury. Nrp2 molecules engineered for increased potency, by oligomerization with an Fc or related strategy, have the potential to be used as selective Sema3-traps. Additionally, molecules specific for certain members of the Sema3 family could potentially be produced by combinatorial approaches using the a1 domain of Nrp1/Nrp2 for specificity in combination with the b1 domain of Nrp2. Taken together, this study demonstrates the mechanism underlying Nrp coagulation factor domain-mediated ligand binding selectivity and advances the search for potent and selective inhibitors of Nrp signaling.

## Supporting Information

Figure S1 **Determination of inter-assay variation.** The ability of Nrp1 and Nrp2 to inhibit VEGF-A binding to Nrp1 affinity plates was measured in three independent trials to determine inter-assay variation. Nrp1 inhibited with an IC_50_ = 1.8 µM (orange line), 1.6 µM (red line), and 2.0 µM (yellow line). The average Nrp1 IC_50_ = 1.8 µM with a standard deviation of 0.2 µM. At the concentrations tested, Nrp2 was unable to fully inhibit VEGF-A binding and therefore only an estimate of the Nrp2 IC_50_ could be made. Nrp2 inhibited with an IC_50_≈310 µM (blue line), ≈120 µM (green line), and ≈160 µM (purple line). The average Nrp1 IC_50_≈200 µM with a standard deviation of 100 µM.
(TIF)
