# Supplementary figures and images for: Mechanism of Selective VEGF-A Binding by Neuropilin-1 Reveals a Basis for Specific Ligand Inhibition
Source: PLoS One. 2012 Nov 8;7(11):e49177. doi: 10.1371/journal.pone.0049177 (PMC3493496; doi:10.1371/journal.pone.0049177)

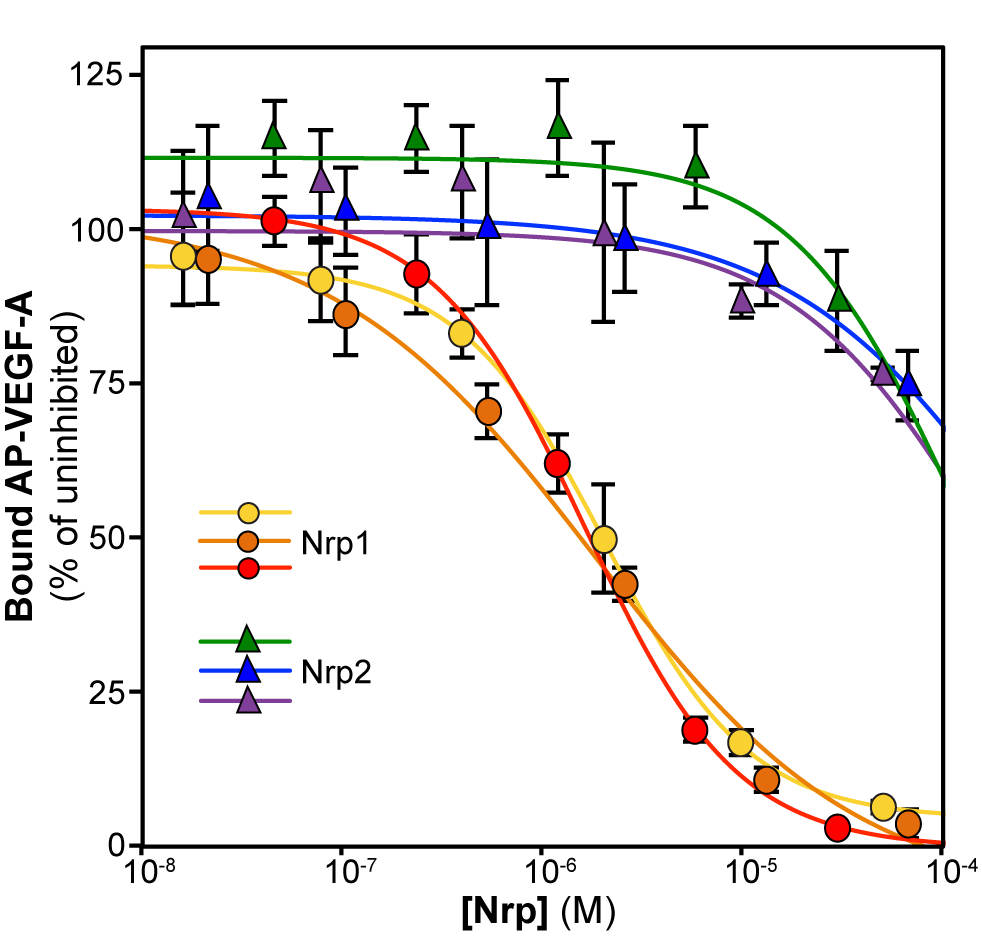

Supplement: Figure S1 — Determination of inter-assay variation. The ability of Nrp1 and Nrp2 to inhibit VEGF-A binding to Nrp1 affinity plates was measured in three independent trials to determine inter-assay variation. Nrp1 inhibited with an IC50 = 1.8 µM (orange line), 1.6 µM (red line), and 2.0 µM (yellow line). The average Nrp1 IC50 = 1.8 µM with a standard deviation of 0.2 µM. At the concentrations tested, Nrp2 was unable to fully inhibit VEGF-A binding and therefore only an estimate of the Nrp2 IC50 could be made. Nrp2 inhibited with an IC50≈310 µM (blue line), ≈120 µM (green line), and ≈160 µM (purple line). The average Nrp1 IC50≈200 µM with a standard deviation of 100 µM. (TIF) [file pone.0049177.s001.tif]
